# Automated Method for Tracking Human Muscle Architecture on Ultrasound Scans during Dynamic Tasks

**DOI:** 10.3390/s22176498

**Published:** 2022-08-29

**Authors:** Saru Meena Ramu, Panagiotis Chatzistergos, Nachiappan Chockalingam, Adamantios Arampatzis, Constantinos Maganaris

**Affiliations:** 1 School of Computing, SASTRA Deemed University, Thanjavur 613401, India; 2 Centre for Biomechanics and Rehabilitation Technologies, Staffordshire University, Stoke-on-Trent ST4 2DE, UK; 3 Department of Training and Movement Sciences, Humboldt-Universität zu Berlin, 10115 Berlin, Germany; 4 School of Sport and Exercise Sciences, John Moores University, Liverpool L3 3AF, UK

**Keywords:** muscle architecture, ultrasonography, in vivo fascicle length, pennation angle, Chan–Vese model, particle filter

## Abstract

Existing approaches for automated tracking of fascicle length (FL) and pennation angle (PA) rely on the presence of a single, user-defined fascicle (feature tracking) or on the presence of a specific intensity pattern (feature detection) across all the recorded ultrasound images. These prerequisites are seldom met during large dynamic muscle movements or for deeper muscles that are difficult to image. Deep-learning approaches are not affected by these issues, but their applicability is restricted by their need for large, manually analyzed training data sets. To address these limitations, the present study proposes a novel approach that tracks changes in FL and PA based on the distortion pattern within the fascicle band. The results indicated a satisfactory level of agreement between manual and automated measurements made with the proposed method. When compared against feature tracking and feature detection methods, the proposed method achieved the lowest average root mean squared error for FL and the second lowest for PA. The strength of the proposed approach is that the quantification process does not require a training data set and it can take place even when it is not possible to track a single fascicle or observe a specific intensity pattern on the ultrasound recording.

## 1. Introduction

The geometric layout of fascicles within a skeletal muscle is known as muscle architecture. The fascicle geometry is primarily defined by two muscle architecture parameters: the fascicle length (FL) between the two aponeuroses and the pennation angle (PA), which is the angle at which the fascicles operate with respect to the longitudinal axis of the muscle. These parameters play important functional roles, impacting on the force-, velocity-, excursion-, and power-generating capabilities of muscle [1,2,3,4].

Advancements in the application of B-mode ultrasonography in the 1990s made it possible to record muscle scans in vivo showing the length and orientation of muscle fascicles at rest or during isometric contraction at different joint angles in those earlier years [1,5,6,7] and during dynamic contractions later on as technological developments allowed higher scanning frequencies [2,3,4,8,9,10,11,12,13,14,15].

Traditionally, ultrasound-based measurements of PA and FL have been made manually [12]. Manual measurements are subjective and time-consuming, especially for the large sequences of ultrasound images recorded during dynamic tasks [4,12,16]. Moreover, manual assessments do not completely exploit the high spatial resolution or temporal resolution in ultrasound images. As a result, localized muscle movements may not be recognized or precisely quantified through this approach [12].

To alleviate the limitations associated with the manual measurement of PA and FL, automated methods using image-processing techniques were developed [3,4,13]. However, the relatively poor image quality and low contrast of ultrasound imaging requires the development of image-processing algorithms that can handle these limitations [17].

Automated quantification of PA and FL has previously been done using one of the following three approaches: feature tracking [12,18,19], feature detection [3,4,13,17], and deep learning [20,21]. Feature tracking involves manually identifying a target fascicle in the first/reference frame of an ultrasound video and tracking this fascicle in subsequent frames using either cross-correlation [19] or the Lucas–Kanade algorithm [18,22]. Since feature tracking relies on the persistence of a predefined target, it requires the targeted fascicle to be visible in all frames without considerable changes in shape or appearance. The loss of the predefined fascicle from the imaging plane or significant changes in its shape or appearance would lead to measurement errors [4,13,17,23]. Even though feature-tracking methods have been successfully used for some years now, the aforementioned conditions are more likely to be met for relatively small and/or slow muscle movements and for more superficial muscles, which are easier to image using ultrasound. The automated analysis of dynamic contractions over a large range of movement and in difficult-to-image muscles remains challenging.

**Figure 1:**
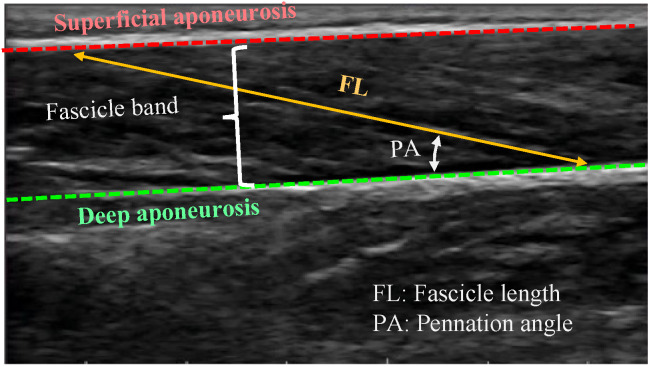
Ultrasound image of gastrocnemius muscle.

Feature detection is based on a specific pattern appearing on all the recorded ultrasound scans. In the case of skeletal muscles, this pattern corresponds to the two aponeuroses appearing as continuous hyperechoic bands and, between them, the fascicles appearing as nonuniformly distributed striations, following a pattern of oblique line-like structures (Figure 1). Feature detection methods detect the dominant fascicle direction and calculate PA and FL based on its intersection with the two aponeuroses. In this case, tracking can be completed as long as the aforementioned distinctive pattern of line-like structures can be reliably identified across the imaging frames. However, this can also become challenging due to the noisy nature of ultrasound imaging, due to imaging artifacts and interference from intramuscular blood vessels, which can obscure critical characteristics of the fascicles [3,4,13].

Lastly, a deep-learning approach uses deep residual and convolutional neural networks for PA and FL estimation [20,21,24]. Although the performance of this approach has been within acceptable error levels, the key common limitation of deep-learning models is that they require a large amount of training data in order to achieve satisfactory results [3,17].

To address the limitations of existing methods, the present study proposes a novel image-processing algorithm that is based on the pattern of movement/distortion within the fascicle band for the automated estimation of PA and FL. The strength of the proposed approach is that the estimation process does not require a training data set and it can take place even when it is not possible to track a single fascicle or observe a specific intensity pattern. Following the introduction of this novel method, its use is demonstrated and compared against previously published results from the literature for the medial gastrocnemius muscle [18]. An assessment of the sensitivity of the results to initialization is also presented.

## 2. Proposed Methodology

**Figure 2:**
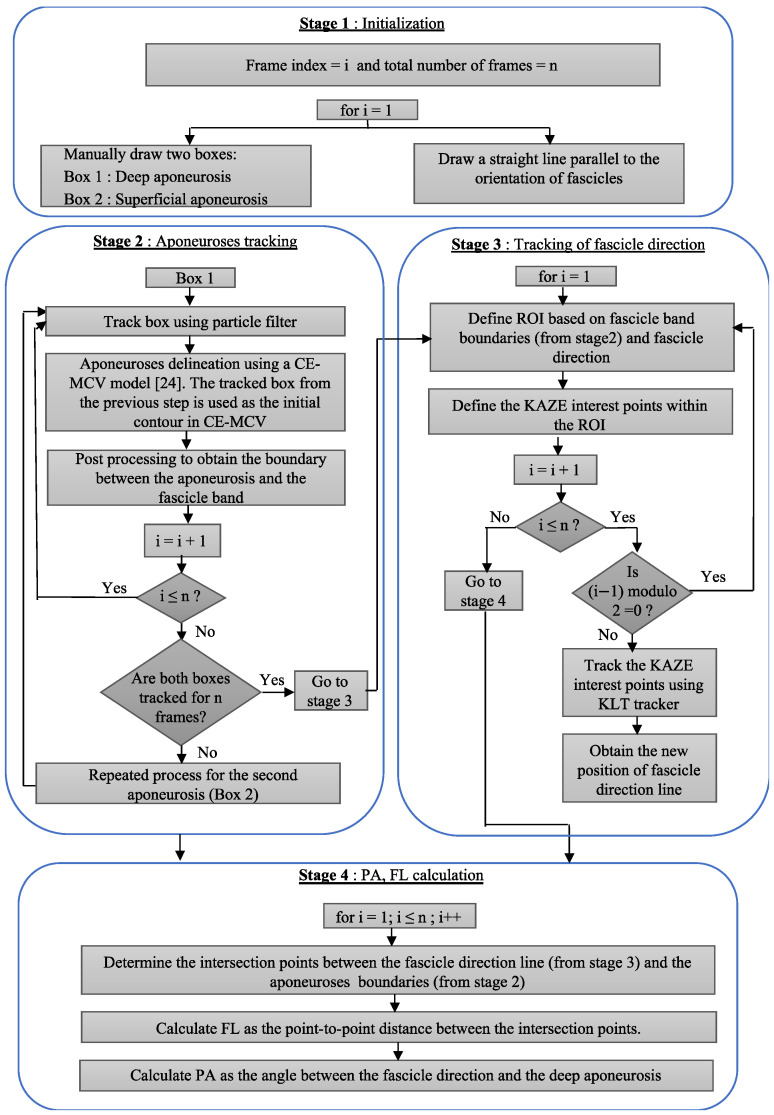
Flow diagram of the proposed approach for estimation of PA and FL.

PA is defined as the angle between the deep aponeurosis and the fascicles. FL is defined as the point-to-point distance between the insertions of the fascicles into the two aponeuroses (Figure 1). Following manual initialization in the first frame of an ultrasound video of a muscle contraction, the proposed approach quantifies changes in FL and PA from the pattern of movement/distortion within the fascicle band and from the changes in the relative distance and orientation of the two aponeuroses. The workflow of the proposed PA and FL estimation algorithm is shown in Figure 2.

The proposed distortion pattern-based image-processing algorithm for the quantification of FL and PA consists of four stages:

**Stage 1:** Initialization
**Stage 2:** Movement tracking of the deep and superficial aponeuroses
**Stage 3:** Tracking of distortion in the fascicle band
**Stage 4:** Calculation of FL and PA

### 2.1. Stage 1: Initialization

**Figure 3:**
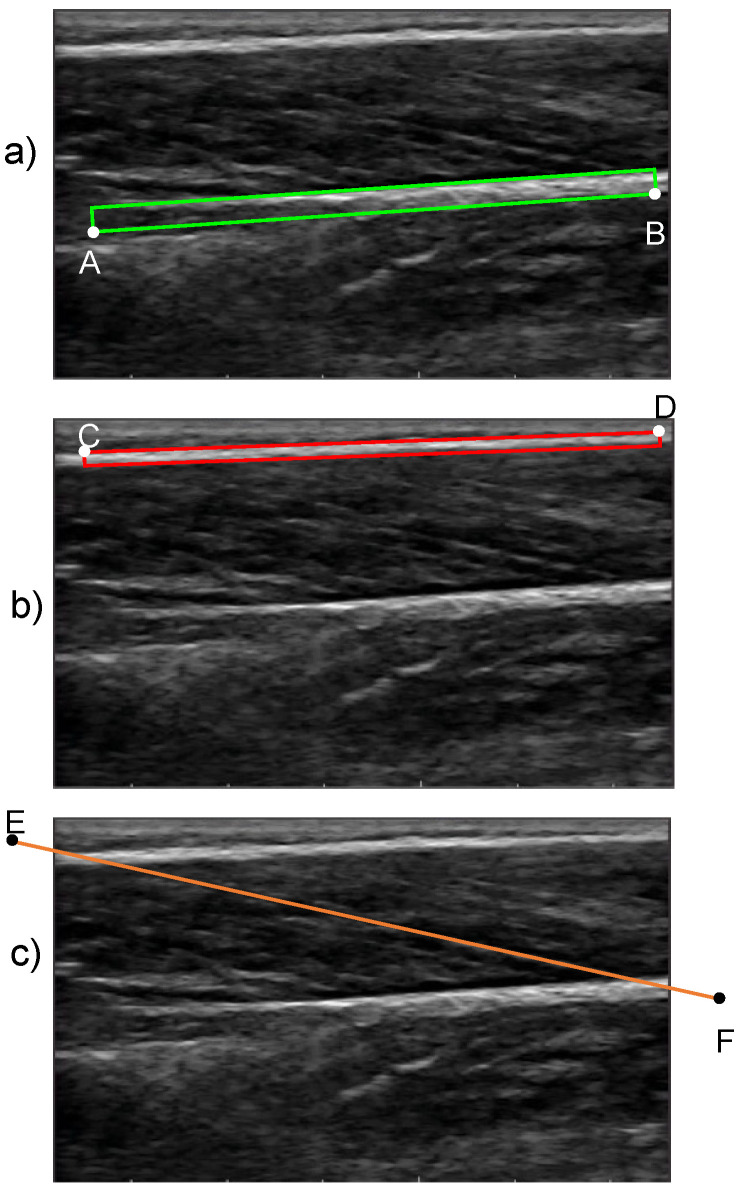
Initialization boxes that enclose (**a**) the deep aponeurosis and (**b**) the superficial aponeurosis. The respective lines that are drawn by the user are denoted as AB and CD for the deep and superficial aponeurosis, respectively. (**c**) The line that is drawn by the user (EF) to initialize the fascicle direction.

The proposed method for the quantification of FL and PA begins with an initialization procedure where the user defines two boxes: one that encloses the superficial and the other that encloses the deep aponeuroses in the reference frame (frame 1). For the deep aponeurosis, starting from the left end, the user defines a line on the bottom boundary of the fascicle band (line AB in Figure 3a) and enters the thickness of the box. Similarly, for the superficial aponeurosis, starting from the left end, the user defines a line on the top boundary of the fascicle band (line CD in Figure 3b) and enters the thickness of the box. The thickness of the boxes was chosen on a trial-and-error basis such that at least ≈90% of the aponeurosis area was enclosed by the initialized boxes. A preliminary sensitivity analysis confirmed that the outcome of the algorithm was not sensitive to the thickness of the initialized boxes (Appendix A). To complete the initialization, the user draws a straight line that is parallel to the direction of fascicles in the fascicle band (line EF in Figure 3c). This line does not need to closely match a specific fascicle. The boxes that enclose the deep and superficial aponeuroses is the input to stage 2, while the fascicle direction line is used as one of the inputs to stage 3.

### 2.2. Stage 2: Tracking of Changes in Superficial and Deep Aponeuroses

**Figure 4:**
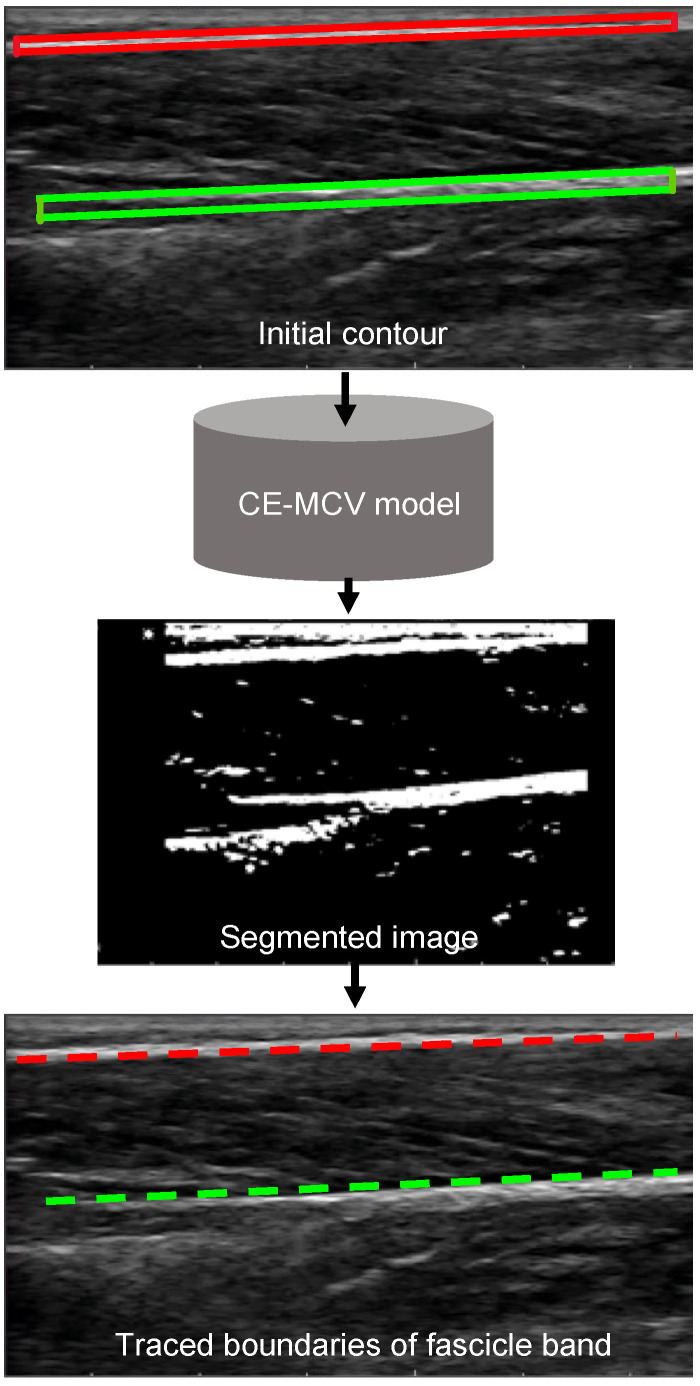
Tracing of (top) (red) and (bottom) (green) boundaries of the fascicle band.

The changes in distance and relative orientation of the two aponeuroses are tracked by frame-wise detection of the top and bottom boundaries of the fascicle band using particle filter in conjunction with a computationally efficient modified Chan–Vese (CE-MCV) model [25]. To this end, the two boxes defined by the user are used as the initial contours for the delineation of the superficial and deep aponeuroses by the CE-MCV model in frame 1. The delineated image is then postprocessed to isolate the boundaries between the aponeuroses and the fascicle band. At the end, the coordinates of the pixels that form the two boundaries are fitted with a straight line to quantify the position and orientation of the two boundaries. The same process is repeated for all subsequent frames. In this case, however, the initial contour used by the CE-MCV model for image delineation is updated for each frame to account for movement between the frames (Figure 4). To this end, the aponeuroses that are enclosed within the user-initialized boxes of frame 1 are tracked for the duration of the video using particle filter. A background of particle filter and CE-MCV algorithms that are used in stage 2 of the proposed method is provided below.

**Particle filter:** The tracking of the aponeuroses is posed as a state estimation problem within a Bayesian inference framework. The goal of the state estimation problem is to estimate the hidden current state x_t_ given the past and the current ultrasound image observations z_1:t_. The optimal estimator $\hat{x_{t}}$ for the state is given by conditional expectation E(x_t_|z_1__:__t_) To obtain the conditional expectation, given all the ultrasound image observations till time(z_1:t_), the posterior probability density function (PDF) of the state x at time t p(x_t_|z_1:t_) has to be estimated and this estimation is done through recursive Bayesian filtering.

The goal of recursive Bayesian filtering is to construct the posterior PDF p(x_t_|z_1:t_), which is obtained through a recursive two stage process: prediction and update. In our problem, since the ultrasound videos are taken during dynamic tasks, the transition and observation models associated with aponeuroses are nonlinear and non-Gaussian in nature, thereby making it difficult to arrive at the closed form solution for the prediction and updating equations of the recursive Bayesian filter. Therefore, the sequential Monte Carlo method (particle filter) was used to approximate the posterior PDF p(x_t_|z_1:t_) [11,26,27,28,29,30,31,32,33,34].

Particle filter is a Bayesian sequential importance sampling technique that is used for estimation of the posterior PDF of the state variable characterizing a dynamic system. Particle filter is a convenient framework for estimating and propagating the posterior PDF of the state variable, regardless of the underlying distribution. Let 𝜗_t_ be the state variable at time t. The state variable characterizes the state of the object, such as position, shape, size, and speed. In our work, the object of interest is either the superficial aponeurosis or the deep aponeurosis, and the state of the aponeurosis is formed by its position (i.e., coordinates of the initialized boxes). The base idea of particle filter is to recursively approximate the posterior PDF of the state variable p(𝜗_t_|z_t_) with a set of weighted particles {𝜗_t_^i^ , a_t_^i^ ; i = 1,2,3…..M}, where 𝜗_t_^i^ is the state variable associated with the i^th^ particle at time t, a_t_^i^ is the weight associated with the state variable of the i^th^ particle at time t, and M is the number of particles. In the present work, since the motion of aponeuroses between two consecutive frames is approximated by affine image warping, the state variable 𝜗_t_ is defined by six affine parameters i.e., 𝜗_t_ = [x_t_, y_t_, *θ*;_t_, 𝑠_t_, 𝑎_t_, 𝜓_t_], where x_t_, y_t_, represent the translation at time t and *θ*;_t_, 𝑠_t_, 𝑎_t_, 𝜓_t_ represent rotation angle, scale, aspect ratio and skew direction at time t, respectively. Each particle represents a hypothesis of the state and is randomly drawn from the prior density. At each time t, particle filter repeats a two-stage process: prediction and update. After a particle is drawn, it is then propagated according to the transition model p(𝜗_t_|𝜗_t−1_) and this forms the prediction stage. Each parameter in 𝜗_t_ is modeled independently by a Gaussian distribution constructed around its counterpart in 𝜗_t−1_. i.e., p(𝜗_t_|𝜗_t−1_) = N(𝜗_t_; 𝜗_t−1_, 𝒫), where 𝒫 is the diagonal covariance matrix whose elements are the variances of affine transformation parameters i.e., 𝓋_x_^2^, 𝓋_y_^2^,. 𝓋_*θ*;_^2^, 𝓋_𝑠_^2^, 𝓋_𝑎_^2^, 𝓋_𝜓_^2^. In the update stage, with the latest measurement (ultrasound image at time t) being available, a weight a_t_^i^ is assigned to each propagated particle in accordance with an observation model that is formed by the observation likelihood p(z_t_|𝜗_t_). In general, the observation models are formed by image features, such as texture, color, gradient, or contours. In the present work, the observation likelihood is formed by the approximation error in sparse representation. The sparse representation of structure of interest is obtained by solving an L1 regularized least squares problem using accelerated proximal gradient descent. The tracking result would be the candidate with the smallest target template projection error. After that, tracking is led by the Bayesian state inference framework, in which particle filter is used for propagating sample distributions over time. Detailed explanation on sparse representation of structure of interest is given in [27,28,29,31,35]. Sparse representation was chosen to handle the occlusion problem, which may arise in ultrasound videos taken during dynamic tasks. After updating, the particles are resampled (sampling with replacement) according to their importance weights to generate an unweighted new particle set, and this step is done to avoid the degeneracy problem (concentration of most of the weights on a single particle) [26,28,29].

The hyperparameters associated with the particle filter framework were number of particles, number of templates in sparse representation, size of the template, variance of affine parameters, Lipschitz constant, regularization constants for target template and trivial template, weighting constant for controlling the energy of trivial templates when occlusion was detected, and maximum number of iterations. All these hyperparameters were set on a trial-and-error basis during preliminary testing and kept constant for all analyses included in this study. For our experiment, the hyperparameters were set as follows: number of particles = 800; number of templates in sparse representation = 10; size of the template (in pixels) = [39, 39]; variance of affine parameters = [0.03, 0.0005, 0.0005, 0.03, 1, 1]; Lipschitz constant = 8; regularization constants for target template and trivial template were 0.2 and 0.001 respectively; weighting constant for controlling the energy of trivial templates when occlusion was detected = 10 and maximum number of iterations = 5.

**Computationally efficient modified Chan–Vese model:** Delineation algorithms based on deformable models are well suited for the analysis of ultrasound images [36,37]. The key idea of deformable-model-based segmentation is that a prior model of the structure of interest is represented either as a 2D curve or as a 3D surface in the image domain and this curve or surface undergoes deformation in an iterative manner to fit onto the boundary of the structure of interest. The deformation field that tells how the model should deform to fit onto the boundary of the structure of interest is obtained by the minimization of an energy functional. Therefore, the segmentation problem is now transformed into an energy functional minimization problem that can be solved with the help of an optimization algorithm. The Chan–Vese (CV) model is one of the most representative and a widely used deformable model [38,39]. The modified CV (MCV) model is a variant of the CV model that is more robust to the type of noise found in ultrasound images, namely the speckle noise [25]. The key limitation of the CV and MCV models is that the computational efficiency of these two models is severely hampered by the use of gradient descent (GD) optimization technique to solve the nonconvex optimization problem of CV and MCV models and the constraint that has been maintained on level set during its evolution. In this regard, the authors in [25] proved that the computational efficiency of the CV and MCV models could be improved by using alternative first-order optimization schemes. Out of the proposed optimization techniques in [25], for the present work, Barzilai–Borwein gradient descent (BB-GD) was chosen. More specifically, the MCV model with BB-GD was chosen from [25] to delineate superficial and deep aponeuroses in each frame of an ultrasound video.

The hyperparameters associated with the CE-MCV model were set either based on previous work by authors of this study [25] or on a trial-and-error basis to produce a satisfactory delineated output, namely a delineated output that contains at least 80% of the subregions in each frame. The weighting constant associated with the length of the curve (𝜌) should be relatively small when the segmentation process targets small individual structures, and it should be relatively large when larger structures or clusters of structure are to be detected [25]. In this regard, 𝜌 was set to 0.0009. The weighting constants associated with data fidelity terms (𝛽_1_ and 𝛽_2_) and area constraint term (𝛾) in the energy functional of the MCV model were set according to the literature [25,40] as one and zero respectively. The stopping criterion threshold (𝜁) was defined as 𝜁 = (0.18)^2^ × dt, where dt is the step size that decides how fast the solution proceeds towards optimum. For our experiment, dt was set to 0.51. The iteration threshold i.e., the maximum number of iterations was set to 5000.

### 2.3. Stage 3: Tracking the Distortion in Fascicle Band

**Figure 5:**
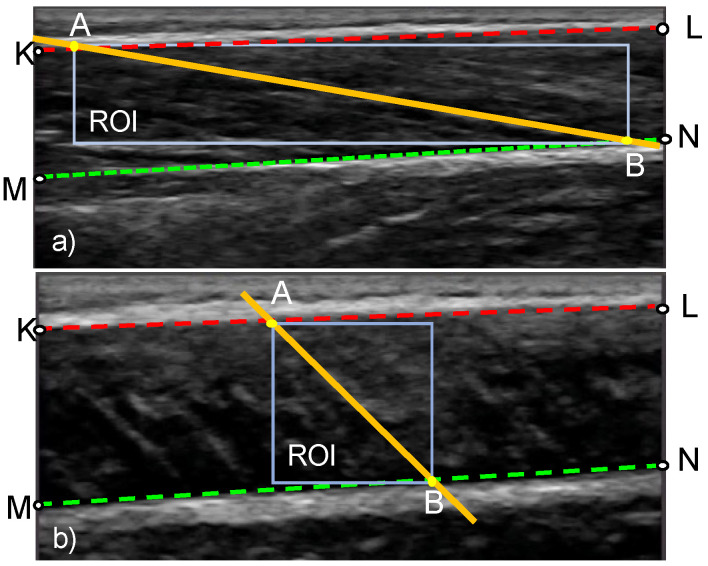
Definition of the region of interest (ROI) in an ultrasound image of a muscle at rest (**a**) and during maximal contraction (**b**). The geometric points that are used in the calculation of FL and PA are also shown. Lines KL and MN, respectively, correspond to the top and bottom boundary of the fascicle band. Points A and B denote the insertion points of the fascicle direction line.

In stage 3, the change in orientation of the fascicles is calculated based on the distortion of a region of interest (ROI) within the fascicle band. The ROI is a rectangle defined by the two insertion points of the fascicle direction line to the aponeurosis lines (points A and B in Figure 5). Once the ROI is defined in the reference frame, the KAZE interest points are identified and tracked in the following frame (tracked frame) using the Kanade–Lucas–Tomasi (KLT) algorithm. To enable the analysis of videos of rapidly changing fascicle direction and length, the ROI is updated and new KAZE interest points defined every two frames throughout the duration of the video (Figure 2). The tracking process is implemented in MATLAB using the pointTracker function. This function uses the optical flow vector (given by the KLT algorithm) to determine the movement of interest points between two frames. Once the new and the old positions of interest points are in hand, the translation (along x-direction and y-direction), rotation, scale (along x-direction and y-direction) and shear/distortion (affine matrix, 6 degrees of freedom) of the ROI is estimated using the estimateGeometricTransfrom2D function in MATLAB. The new position of the fascicle direction line in the tracked frame is found by applying the affine matrix given by the estimateGeometricTransform2D function over the coordinates of the fascicle direction line in the reference frame. Once the analysis of the first two frames is complete, the tracked frame becomes the new reference frame and the entire process is repeated (Figure 2).

The hyperparameters of the MATLAB functions for finding the KAZE interest points in the reference frame, tracking the defined KAZE interest points, and estimation of 2D geometric transformation matrix between two frames were chosen on a trial-and-error basis during preliminary testing and they were kept constant for all analyses included in this study. The following paragraphs give a brief description of KAZE interest points and the MATLAB functions pointTracker and estimateGeometricTransform2D.

**KAZE interest points:** An image could be represented in an abstract manner by detecting features (a.k.a. interest points) at different scale levels (multiscale image processing) and associating a local descriptor with each of the detected features. The existing approaches for multiscale feature detection and description use linear scale space (Gaussian scale space, which is one instance of linear diffusion). However, the limitation of Gaussian blurring is that it smoothens the details and noise in the image to the same level while evolving the image through scale space, thereby reducing the localization accuracy and distinctiveness. To alleviate the limitation of linear scale space, the authors in [41] proposed a 2D feature detection and description approach called KAZE that operates completely in a nonlinear scale space. The nonlinear scale space was constructed using additive operator splitting techniques and nonlinear diffusion (variable conductance diffusion). Diffusion was calculated according to Weickert conductivity coefficient (diffusion type: “edge”) [41]. The strength of nonlinear scale space is that at each scale level, the noise is removed without disturbing the image details, thereby improving the localization accuracy and distinctiveness.

The hyperparameters associated with the MATLAB function for finding the KAZE interest points in the reference frame are: (1) rectangular ROI, specified as a row vector of the form [x, y, width, height] where x, y denotes the x-coordinate and y-coordinate of the starting point of the rectangle (top left corner) (Figure 5), width and height denote the width and height of the rectangle respectively (2) number of scale levels and number of octaves for multiscale analysis and (3) method to compute diffusion (conductivity). To enable multiscale analysis, the number of octaves should be an integer that is greater than 1 and more features can be detected when the number of octaves is more. Similarly, the number of scale levels is an integer in the range [3, 10] and smooth-scale changes can be achieved if the number of scale levels is high. For our experiment, the rectangular ROI was defined as [A_x_, A_y_, width = B_x_ − A_x_, height = B_y_ – A_y_], where A_x_ and B_x_ are the x-coordinates of points A and B respectively (Figure 5); A_y_ and B_y_ are the y-coordinates of the points A and B respectively (Figure 5); the number of octaves and number of scale levels were set to 9 and 5 respectively.

**pointTracker:** The pointTracker function in MATLAB is used to track the KAZE interest points defined in the reference frame. Fundamentally, this function uses the KLT algorithm to calculate the optical flow of a region to determine the movement of structure of interest between two frames. The hyperparameters associated with pointTracker function are: (1) region of size m×m around each interest point (block size) (2) number of levels in the image pyramid for multiresolution tracking (3) maximal bidirectional error and (4) maximum number of iterations required to obtain the optimum solution. For our experiment, block size, number of levels in image pyramid, maximal bidirectional error, and maximum number of iterations were set to 5 mm × 5 mm, 9, 3 mm, and 40, respectively. The output of this function is the new location of KAZE interest points defined in the reference frame.

**Figure 6:**
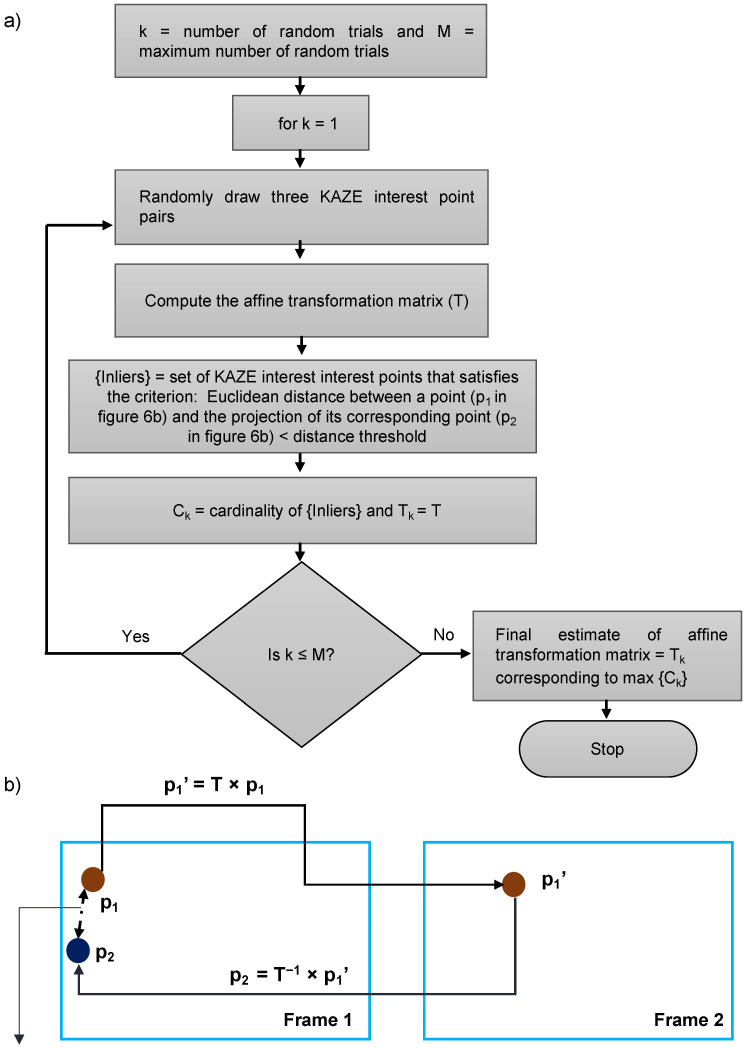
(**a**) Flow diagram for estimation of affine transformation matrix using M-SAC algorithm in estimaGeometricTransform2D MATLAB function (**b**) Illustration of maximum distance threshold.

**estimateGeometricTransform2D:** This MATLAB function estimates the 2D geometric transformation matrix between two frames by mapping the inliers in KAZE interest points from the reference frame to the inliers in KAZE interest points from the tracked frame. In the process of finding the corresponding interest points between two frames, it is necessary to determine the interest points that are true matches (inliers) and the interest points that are not matching (outliers). Random sample consensus (RANSAC) is most commonly used for this purpose and many variants of RANSAC have been proposed in the literature, out of which the M-estimator sample consensus (MSAC) variant is used by this MATLAB function. RANSAC and its variants are used for estimating the best-fit model from the data especially when the data contain a large number of outliers [42]. Here, geometric transformation over the image is considered as the model and the task is to estimate the model (geometric transformation matrix) from the data (a pair formed by the old position and the new position of the KAZE interest points). Each model has a minimal set (i.e., smallest number of interest-point pairs) from which the model can be computed. The sequence of steps involved in finding the best-fit model is depicted in Figure 6a. The hyperparameters associated with this function are: (1) maximum random trials, (2) type of geometric transform, and (3) maximum distance (in pixels) between a point and the projection of its corresponding point (Figure 6b). For our experiment, the type of geometric transform was set to affine, the maximum number of random trials and maximum distance between a point in reference frame and the projection of its corresponding point from the tracked frame were set to 1,000,000 and 30 pixels, respectively. Since the type of geometric transformation was set to affine, for the computation of affine transformation matrix, the minimal set should consist of three pairs of KAZE interest points.

### 2.4. Stage 4: Calculation of PA and FL

After obtaining the new position of the fascicle direction line and the boundaries of the fascicle band, the insertion points of the fascicle direction line (points A and B in Figure 5) are determined, followed by the computation of FL as their Euclidean distance. PA is calculated as the angle between the fascicle direction line and the deep aponeurosis (angle ABM in Figure 5).

## 3. Demonstration of the Proposed Approach

The proposed concept was tested on normative previously published ultrasound data for the medial gastrocnemius muscle during isokinetic ankle plantar flexion contractions at 30, 120, 210, and 500 deg/s [18]. Twelve videos were analyzed in total (three videos per plantar flexion speed), each including a single contraction. Manual measurements of PA and FL were performed on specific frames of the isokinetic videos (6 frames per video) by an expert [18]. The automated measurements of PA and FL using the proposed approach were compared against these published ground-truth measurements.

Moreover, to get an assessment of relative performance of the proposed method against literature, an established feature tracking method [18] and an established feature detection method [13] were also used to analyze the same videos. The relative performance of the three methods was assessed based on their degree of closeness to the ground-truth measurements.

To support the repeatable use of the proposed methodology, the initialization of the fascicle direction line (Line EF, Figure 3c) was done following a predefined process. More specifically, the user was instructed to draw the fascicle direction line along a clearly visible fascicle and then to move it to the center of the image, ensuring that the two intersection points between the fascicle direction line and the two aponeuroses (points A,B Figure 5) were within the ultrasound image. The sensitivity of results to the placement of the initialized fascicle direction line and to small deviations in the initial PA was assessed in a final series of tests.

### 3.1. Comparison against Manual Measurements and Established Methods from Literature

**Figure 7:**
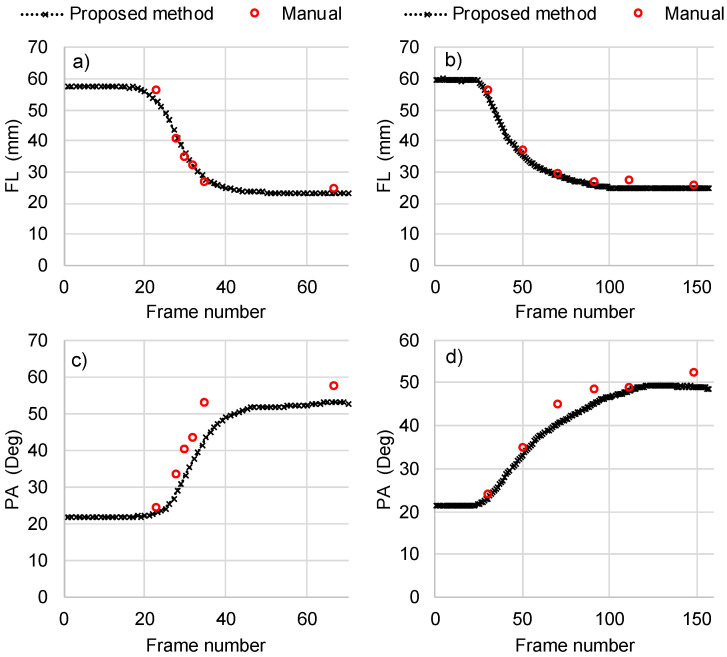
Comparison between the automated calculations using the proposed algorithm and manual measurements of fascicle length (FL) (**a**,**b**) and pennation angle (PA) (**c**,**d**). Results are presented separately for the contraction where the difference between computerized and manual measurements was the highest (**a**,**c**) and for a contraction where it was the lowest (**b**,**d**). More specifically, graphs (**a**,**c**) correspond to an ankle plantar flexion contraction at 500 deg/s, graphs (**b**,**d**) to ankle plantar flexion at 30 deg/s. For comparison, the respective root mean square errors (RMSE) between computerized and manual measurements are also shown.

Manual measurements were available for six frames for each of the analyzed contraction videos. The level of agreement between manual and automated measurements was evaluated using root mean squared error (RMSE) and the coefficient of variation (CoV). Average RMSE for FL was equal to 2.7 mm, 3.4 mm, 1.5 mm, and 3.1 mm for contractions at 30, 120, 210 and 500 deg/s respectively. For the same contraction speeds, RMSE was 3.3 deg, 3.7 deg, 5.6 deg, and 5.2 deg for PA. Results for a video with lowest RMSE and for the one with the highest RMSE are presented in Figure 7 to provide a sense of the physical meaning of low and high RMSE in the context of this comparison.

The CoV between manual measurements and the automated calculations of the proposed method was 4% and 7% for FL and PA, respectively. This level of variation was deemed to be satisfactory. According to the literature, the CoV for FL and PA measurements in gastrocnemius muscle across measurements should be ≤10% [18,43].

When the same images were analyzed using an established feature tracking method [18] and an established feature detection method [13] from the literature, the proposed approach achieved the lowest average RMSE for FL followed closely by feature tracking (Table 1). Moreover, it achieved the second-lowest average RMSE for PA. In both cases, the average RMSE of feature detection was relatively larger than the other two methods.

All the three automated methods achieved CoVs lower than the predefined threshold of 10% [18,43] for FL (Table 1). For PA, only the proposed approach and feature tracking achieved CoV < 10%. More specifically, the CoV between the manual and automated measurements of FL was 4% for the proposed approach and feature tracking, while the feature detection approach achieved a CoV of 9%. With respect to PA, the CoV between manual and automated measurements was 7%, 5%, and 16% for the proposed approach, feature tracking and feature detection respectively.

### 3.2. Sensitivity to Initialization

The video that gave the highest RMSE relative to manual measurements (Figure 7a,c) was used to assess the effect of initialization on the calculations. To this end, the analysis was repeated for different initializations of the oblique fascicle direction line and the difference in the calculated FL and PA relative to the reference initialization and to manual measurements were assessed. Six different scenarios were tested in total: four scenarios for different placements of the fascicle direction line without any change in the initial PA and two cases where initial PA was slightly altered. Different placements were produced by moving the points E, F of the reference initialization (Figure 3c) by 10, 20 pixels to the right and 10, 20 pixels to the left. This resulted to parallel displacements of 0.9 mm, 1.8 mm, −0.9 mm and −1.8 mm, respectively from the reference position. A small rotation was imposed by moving point E 10 pixels in one direction and point F by 10 pixels to the opposite direction, thus changing the initial PA by ±1.4 deg.

#### 3.2.1. Parallel Shift of Fascicle Direction Line

**Figure 8:**
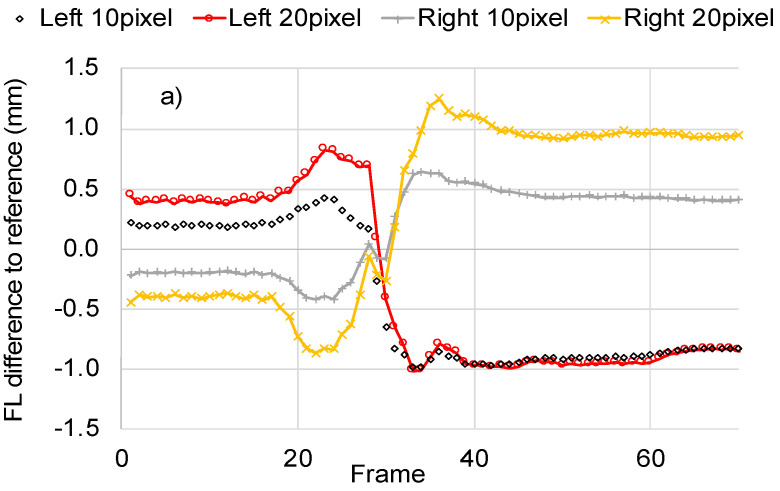

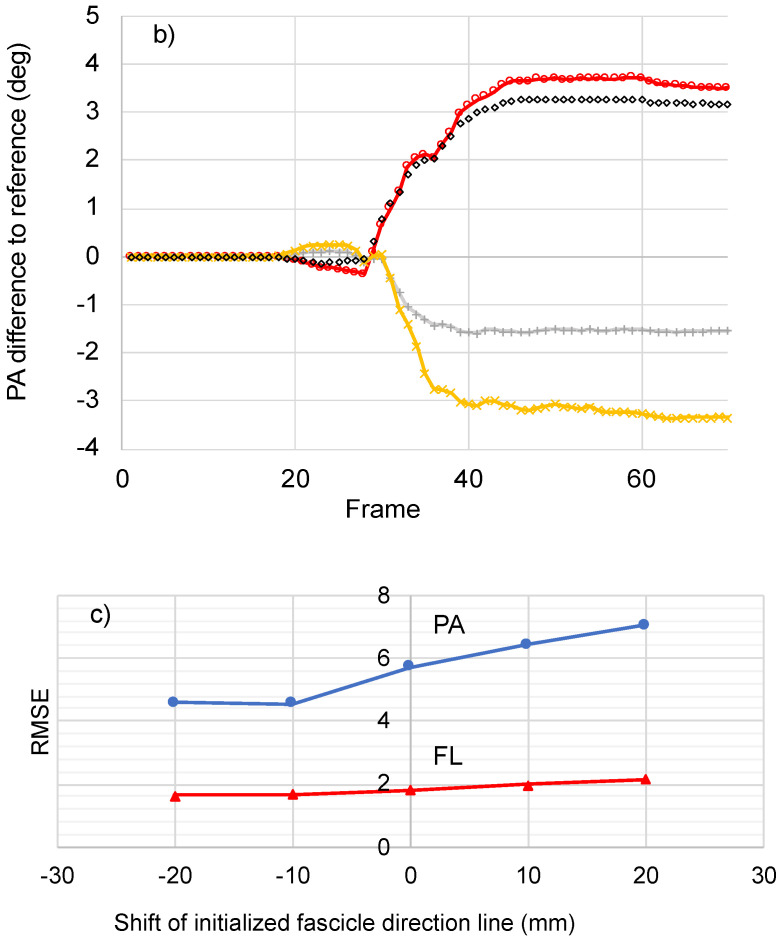
The sensitivity of FL and PA to the initial placement of the fascicle direction line initialization. Different initialization scenarios are generated by shifting the reference initialization line to the left (−10, −20 pixels) or to the right (10, 20 pixels) from the center of the image. The resulting difference in FL (**a**) and PA (**b**) relative to the reference initialization is presented for all frames of a contraction video (Figure 7a,c). The effect on accuracy is demonstrated based on the RMSE against ground truth for FL (red line) and for PA (blue line) (**c**).

The results indicated that a parallel shift of the initialized fascicle direction line to the right or to the left from the center of the image (i.e., reference position) led to differences in the calculations, which changed as the contraction progressed. For example, a parallel shift of the fascicle direction line to the right by 20 pixels (1.8 mm) led to an underestimation of FL of 0.4 mm for the first frame (−0.8% difference to reference) and to an overestimation of FL of 0.9 mm (4% difference to reference) for the last frame (Figure 8a). Parallel shift to the left had the opposite effect, leading to initial overestimation and finally to an underestimation of FL for maximum contraction relative to the reference initialization. As expected, parallel shift of the initialized fascicle direction line did not change the initial estimation for PA. However, it led to differences of up to 4 degrees for maximum contraction.

Interestingly, the response of the algorithm seems to stabilize when the initialization line is moved to the left of the image. As can be seen in Figure 8a,b, a shift by 10 and 20 pixels to the left leads to very similar calculations for maximum contraction regardless of the initial differences in PA and FL for the muscle at rest. This pattern of convergence is also evident when the computerized calculations are compared against ground truth. As can be seen in Figure 8c, shift to the left of the initialization line by 10 or 20 pixels led to very similar and significantly reduced RMSE for PA and FL. These findings indicate that optimum initialization for maximum accuracy and robustness might be possible.

#### 3.2.2. Initial PA

**Figure 9:**
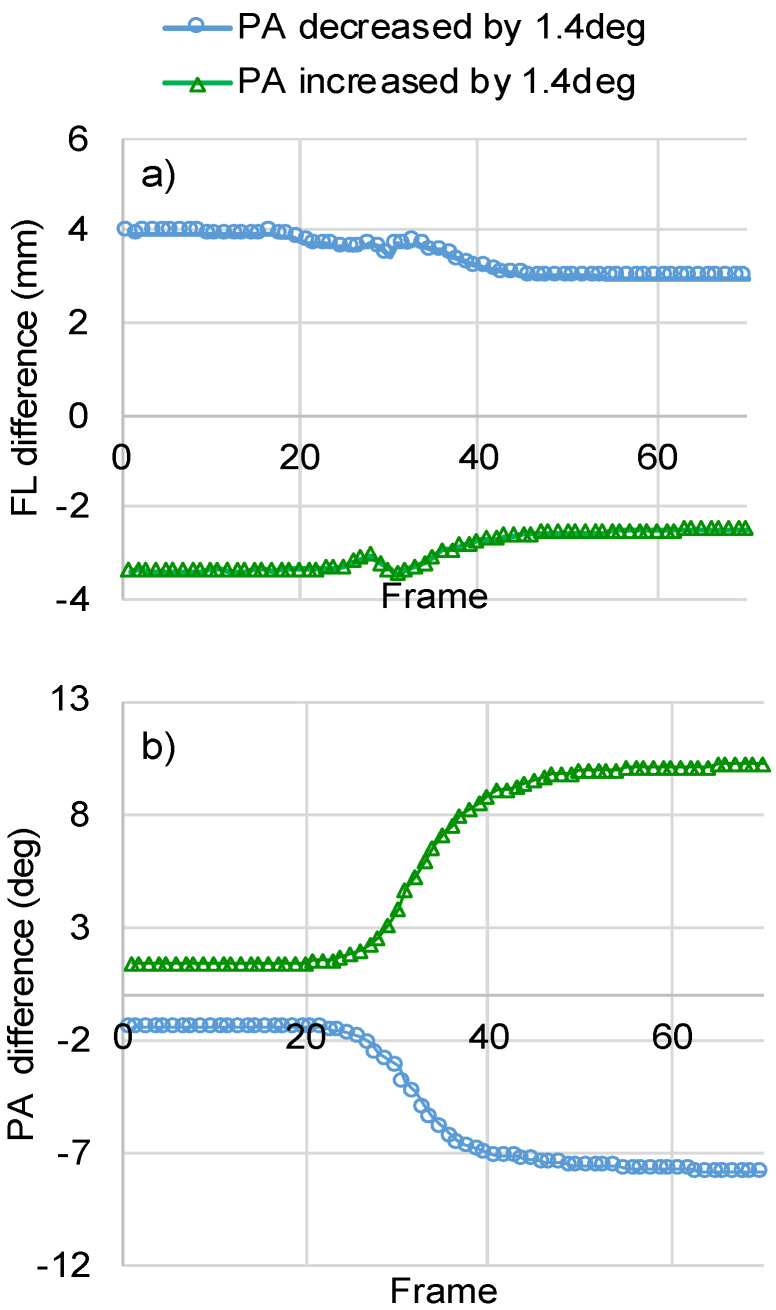
The sensitivity of FL and PA to small changes in the initialized PA. Different initialization scenarios are generated by rotating the reference initialization line to the left (1.4 deg reduction in initial PA) or to the right (1.4 deg increase in initial PA). The resulting difference in FL (**a**) and PA (**b**) relative to the reference initialization is presented for all frames of a contraction video (Figure 7a,c).

Small deviations in the orientation of the initialization line appear to cause substantial changes in FL and to changes in PA that are magnified over the duration of the contraction. Indicatively, increasing the PA during initialization by 1.4 deg (−6.4% difference to reference) decreased the calculated FL for the at-rest muscle in frame one by 3.9 mm (−6.8% difference to reference). At the end of the contraction video, this difference was 3.0 mm (−13% difference to reference) (Figure 9a). With regards to PA, 1.4 deg underestimation at initialization was magnified to 7.8 deg underestimation (−14.8% difference to reference) of PA at the final frame of the video (Figure 9b).

The sensitivity of the calculations to the PA initialization has a strong influence on the accuracy of the method. Rotating the initialization line from what the user considered to be in alignment with the direction of the fascicles significantly increased the difference of FL to ground truth. More specifically, the RMSE for FL increased from 1.8 mm to 3.9 mm and 3.7 mm for 1.4 deg decrease and increase of the initialized PA, respectively. RMSE for PA was also significantly affected by initialization. In this case, however, the effect appeared to us monotonous. More specifically, 1.4 deg reduction in PA during initialization increased the RMSE to 10.1 deg (from 5.7 deg for reference), while increasing the initialization PA by the same amount decreased the RMSE to 2.9 deg (from 5.7 deg for reference).

## 4. Discussion

Traditionally, FL and PA have been measured manually. Manual measurements are subjective and time-consuming, especially for the large sequences of ultrasound images recorded during dynamic tasks. To alleviate the limitations associated with manual measurements of PA and FL, automated methods using image-processing techniques were developed [3,12,13,18]. Previous studies on automated methods for the quantification of FL and PA rely on the presence of a single user-defined fascicle (feature tracking) or on the presence of a specific intensity pattern (feature detection) across the recording or on a large manually analyzed training data set (deep-learning approach). The need for a fascicle or a pattern to be visible across the recording can undermine the accuracy of FL and PA measurements in ultrasound videos taken during dynamic movements or for deeper muscles, which are more difficult to image. Deep-learning approach is not affected by these issues, but their applicability is restricted by their need for large training data sets [3].

To address the limitations of the existing methods for automated measurements of FL and PA, the present study proposes a novel method that tracks changes in FL and PA based on changes in the position and orientation of the two aponeuroses and the movement/distortion pattern within the fascicle band. The strength of this method stems from its ability to utilize information on movement from every trackable point within the ROI and not only from points that belong to a specific fascicle or pattern. As a result, it can be applied to recordings where it is impossible to track a single fascicle or pattern. The only key requirements for its use are: (a) that the two aponeuroses are visible across the recording and (b) that the fascicle direction can be inferred in at least one frame for initialization. In its current form, the proposed algorithm in this study used linear curve-fitting to calculate the boundaries between the fascicle band and the two aponeuroses. In the future, linear curve fitting could be easily replaced by polynomial fitting to enable also the study of muscles whose aponeuroses do not appear as straight lines in ultrasound imaging.

The performance of the proposed approach was assessed against (1) manual measurements of FL and PA and (2) PA and FL results obtained using alternative automated methods available in the public domain [18]. The method of [18] was included in this study as a representative method that is based on feature tracking. They compared the accuracy of their method against Ultrack, a common tool for the tracking of FL and PA, and concluded that for the same initialization, their method could achieve better accuracy than Ultratrack [18]. The method by [13] was included as a representative method for feature detection. In [13], the image of the region of interest between the two aponeuroses is first converted into a binary image using quantile thresholds of the image intensities, followed by detecting possible fascicle snippets by the bwtraceboundary function of MATLAB. The semiautomatic approach allows for setting also manual snippets, which was not done for this study.

The results indicated a satisfactory level of agreement between manual and automated measurements of FL and PA using the proposed method. When compared against automated methods for the measurement of FL and PA (feature tracking and feature detection approaches), the proposed method achieved the lowest average RMSE for FL and the second lowest for PA. In both cases, the CoV was well below 10%, the threshold specified in literature as acceptable. The observed relatively lower accuracy for PA could be due to errors in identifying the angle of the fascicles (fascicle direction line orientation) accurately in the initialization phase. The proposed algorithm calculates FL and PA as changes from the reference values that are calculated during initialization based on input by the user. This approach enables the creation of a method that is robust to the disappearance of specific fascicles or intensity patterns in subsequent frames. At the same time, this can also make the method sensitive to initialization. In the case of the two aponeuroses, initialization is only the first step for the detection of the boundaries of the two aponeuroses based on image delineation and tracking. In this case, the only prerequisite for correct initialization is that the user needs to create boxes that enclose the two aponeuroses. This process is made significantly easier by the fact that the two aponeuroses appear as areas of high contrast in ultrasound imaging. The operator-independent nature of initialization with regards to the two aponeuroses is also supported by a preliminary analysis (Appendix A) indicating that the results were not affected by the thickness of two initialized boxes (Figure 3a,b).

On the contrary, the results appear to be sensitive to the initialization of the fascicle direction line. Based on the results presented here, this seems to be a bigger problem for PA than FL (Figure 8 and Figure 9). Because of trigonometry, small variations in FL during muscle contraction might result in large variations in PA [18]. Moving forward, this challenge could be addressed by adding an additional step into the process for the operator-independent initialization of fascicle direction in the reference frame. The feature detection approach of [13] appears to be a very good candidate method for this purpose. A second dimension of sensitivity to initialization that was not explored here is the selection of the reference frame. In this study, all calculations were conducted based on an image of the muscle at rest. The criteria dictating optimal selection of reference for maximum accuracy should also be explored in the future.

## 5. Conclusions

The purpose of this study was to demonstrate the idea that the pattern of movement and distortion of muscle fascicle band could be used to quantify PA and FL in the ultrasound videos taken during activities in which the length of the muscle changes. The proposed approach was demonstrated with ultrasound videos of medial gastrocnemius muscle taken during isokinetic contractions. The results indicated a satisfactory level of agreement between the manual and automated (proposed approach) measurements of FL and PA. When compared against the established feature tracking and feature detection methods from the literature, the proposed method achieved the lowest average RMSE for FL and the second lowest for PA. The proposed method offers the advantage that the quantification process does not require a training data set and it can take place even when it is not possible to track a single fascicle or observe a specific intensity pattern on the ultrasound recording. However, in its current form, the performance of the proposed approach is sensitive to the fascicle direction line initialization. Sensitivity to initialization can have a detrimental effect on the accuracy and on the time efficiency of the method and should be addressed for the development of a widely applicable automated method. Combining the concept presented here with established methods capable of objectively identifying the dominant direction of fascicles could address this limitation.

## Figures and Tables

**Table 1:** Coefficient of variation (CoV) and average root mean square error (RMSE) between manual and automated measurements of fascicle length (FL) and pennation angle (PA). Results for the proposed distortion-based approach are compared against feature detection and feature tracking methods from literature.

	FL		PA	
	CoV	RMSE (mm)	CoV	RMSE (deg)
**Distortion-based approach**	4%	2.4 ± 1.3	7%	4.4 ± 1.4
**Feature tracking approach** [18]	4%	2.7 ± 1.3	5%	3.3 ± 1.7
**Feature detection approach** [13]	9%	5.5 ± 2.6	16%	8.8 ± 2.4

**Figure A1:**
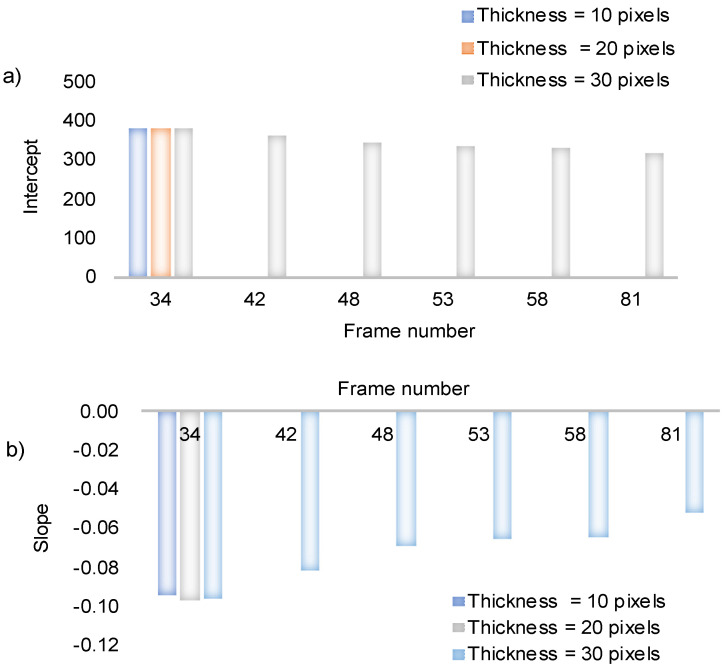
Box sensitivity analysis for deep aponeurosis (**a**) Slope of bottom boundary of fascicle band for different thickness of the box around deep aponeurosis and (**b**) Intercept of bottom boundary of fascicle band for different thickness of the box around deep aponeurosis.

## Data Availability

The ultrasound videos (medial gastrocnemius muscle taken during isokinetic contractions) analyzed in this study have been made publicly available by the authors of [18] (J. F. Drazan, T. J. Hullfish, and J. R. Baxter, “An automatic fascicle tracking algorithm quantifying gastrocnemius architecture during maximal effort contractions,” PeerJ, vol. 7, pp. e7120–e7120, July 2019, doi: 10.7717/peerj.7120).

## Appendix A

**Box sensitivity test for tracing of bottom and top boundaries of fascicle band:**

For the deep aponeurosis, the thickness of the box was chosen as 20 pixels. To check whether the tracing of bottom boundary of the fascicle band was sensitive to thickness of the box that encloses ≈90% of the deep aponeurosis, a sensitivity test was carried out with thickness of the box being defined as thickness = {20 ± (50% of 20)} pixels. The test was carried out on a single ultrasound video. More specifically, the six frames that were defined for validation part were analyzed. For each thickness of the box, the slope and intercept of the line that defines the bottom boundary of the fascicle region were noted for each frame. From Figure A1a,b, it is evident that the slope and intercept of the line that defines the bottom boundary of the fascicle band were insensitive to changes in the thickness of the box (±10 pixels change relative to 20 pixels) initialized around deep aponeurosis in frame 1 of the ultrasound video.

The same type of analysis was done for superficial aponeurosis too. For the superficial aponeurosis, the thickness of the box was chosen as 10 pixels. To check whether the tracing of top boundary of the fascicle band was sensitive to the thickness of the box that encloses ≈90% of the superficial aponeurosis, a sensitivity test was carried out with thickness of the box being defined as thickness = {10 ± (50% of 10)} pixels. From the analysis, it was found that slope and intercept of the line that defines the top boundary of the fascicle band were insensitive to the changes in thickness of the box (±5 pixels change relative to 10 pixels) initialized around superficial aponeurosis in frame 1 of the ultrasound video.
